# Investigation of flow state occurrence during robotic virtual reality operations

**DOI:** 10.1038/s41598-025-24215-0

**Published:** 2025-11-19

**Authors:** Uwe Bieri, Melanie Baumgartner, Anne-Raphaëlle Richoz, Daniel Eberli, Cao Tri Do, Cédric Poyet

**Affiliations:** 1 Department of Surgery, Division of Urology, Cantonal Hospital Baden, Baden, Switzerland; 2 Department of Psychology, University of Fribourg, Fribourg, Switzerland; 3 Department of Urology , University Hospital of Zurich, University of Zurich, Zurich, Switzerland; 4 Translational Neuromodeling Unit, Institute of Biomedical Engineering, University of Zurich & ETH Zurich, Zurich, Switzerland; 5 Department of Urology, City Hospital Triemli Zurich, Zurich, Switzerland

**Keywords:** Flow state, EEG, Theta activity, Heart rate, Surgeons, Neurofeedback, Biofeedback, Performance, DaVinci skills simulator, Neuroscience, Cognitive neuroscience, Attention, Psychology, Human behaviour

## Abstract

Flow is the optimal mental state for peak focus and performance, positively correlating with well-being. This study investigated electroencephalographical (EEG) and circulatory heart rate (HR) markers of flow during virtual reality (VR) surgical training on the da Vinci Skills Simulator. Twenty urologic surgeons participated, categorised as High or Low Performers based on VR task scores. Generalised estimating equations and Spearman’s rank correlation were used to analyse differences in EEG, HR, Flow Short Scale scores, and professional experience. High performers exhibited significantly higher Theta activity *Wald*(1) = 5.96, *p* = .015; *Wald*(1) = 5.11, *p* < .001) and a positive correlation between VR scores and flow *r*(10) = 0.63, *p* = .048). No significant HR differences were observed. The study highlights the importance of Theta activity in achieving flow, suggesting that neurofeedback and biofeedback could provide a point of departure to enhance surgical performance and well-being of surgeons and patient health.

**Supplementary Information:**

The online version contains supplementary material available at 10.1038/s41598-025-24215-0.

## Introduction

In the dynamic and demanding field of surgery, attaining a state of optimal performance is not only a pursuit of excellence but a necessity. The concept of “Flow,” a term coined by psychologist Mihaly Csikszentmihalyi in the 1970s^1^, has sparked considerable interest in various disciplines for its potential to enhance performance and well-being. Flow represents a state of heightened focus and immersion, where an individual’s skills align perfectly with the challenges at hand, leading to peak performance and personal fulfilment^2^. While extensively studied in sports^3^, music^4^, and video gaming^5^, investigating flow within surgical practice remains relatively unexplored.

Neuroimaging studies, particularly those utilising electroencephalography (EEG), have significantly advanced the understanding of the Flow State. EEG measures electrical activity in the brain and is valuable for investigating different states of alertness and focus^6,7^. Research has shown that specific EEG patterns are associated with the Flow State. Increased Theta wave activity, particularly in the frontal and central regions, is a consistent finding in Flow research^8^. Theta waves (4–8 Hz) are linked to cognitive control and high engagement, indicating a state where the brain efficiently processes information and maintains focus^9,10^. For instance, during tasks that induce flow, such as mathematical problem-solving or gaming, participants exhibit higher Theta power, reflecting deep concentration and cognitive engagement^11^. These findings suggest that Theta activity may be a neural marker for flow, providing a measurable indicator of this optimal state. Frontal midline Theta activity, originating from the anterior cingulate cortex (ACC), is particularly relevant to flow. The ACC plays a crucial role in attention, cognitive control, and emotional regulation, all essential for achieving flow^12^. Studies have shown that higher frontal midline Theta activity is associated with better performance in tasks requiring sustained attention and precision^13,14^.

Neurofeedback, a technique that trains individuals to regulate their brain activity, has been used to enhance flow experiences. By providing real-time feedback on EEG patterns, individuals can learn to increase Theta activity, facilitating entry into the Flow State^15^. Studies have demonstrated that neurofeedback can improve performance and reduce anxiety in musicians and athletes, suggesting its potential for broader applications^16,17^. In surgical training, real-time neurofeedback protocols targeting Theta activity while performing robotic surgical tasks have not yet been investigated.

Heart rate (HR) monitoring is an additional physiological measure used to study flow^18^. A lower HR is typically associated with relaxation and reduced anxiety, both conducive to achieving flow. Studies have found that during Flow-inducing activities, such as gaming or music performance, individuals exhibit lower HR, indicating a state of calm concentration^18,19^. In surgical training, the heart rate is established as a biomarker for stress^20^ but this has not been investigated in the context of flow state.

The high-stakes nature of surgical performance makes flow particularly valuable. Surgeons must perform under immense pressure, and being in the flow state may help them maintain high focus and precision, reducing errors and improving patient outcomes, which makes investigating flow states critically important. Furthermore, the high rates of burnout and stress among surgeons highlight the need for strategies that can enhance their performance and well-being^21^. Advances in minimally invasive techniques have led to increased use of robotic surgery systems like the da Vinci Surgical System, which can help improve performance by offering enhanced precision and dexterity, although they require specialised training^22^. Virtual reality (VR) simulators, such as the da Vinci Skills Simulator (dVSS), provide a safe and controlled environment for surgeons to improve their skills^23^. VR simulations allow for repeated practice and allow one to study the factors influencing surgical performance under various and controlled conditions.

The current study aimed to investigate the use of EEG and HR as objective biomarkers of flow state among urological surgeons in a controlled surgical environment. This first step in understanding and harnessing the flow state seeks to provide foundations for developing training programs and neurofeedback protocols that may not only improve surgical outcomes but also enhance the quality of life for surgeons.

## Methods

### Sample size

The sample size was estimated for conducting a mixed analysis of variance (ANOVA) involving a between-subjects factor (performance group: High Performers vs. Low Performers) and a within-subjects factor (measurement across three rounds). A medium-sized effect for the between-subjects factor (*f* = 0.30) and a 0.50 correlation between levels of the within-subjects factor were estimated based on standard practices in power analysis for studies of this nature^24^. An alpha level of 0.05 with a power of 0.80 was set and calculated using G*Power 3.1^25^. The required sample size was estimated to be 20.

### Participants

21 urologic surgeons (aged 26–40 years; 5 females; *M* = 31.05, *SD* = 4.77) from the University Hospital Zurich (USZ) were recruited. One participant dropped out during the study process, resulting in a final sample of 20 surgeons. This study was approved by the local ethics committee (BASEC-NR. 2021 − 0158) and conducted in accordance with the approved protocol. All methods were performed in compliance with the relevant guidelines and regulations, including Swiss legal requirements, the principles of the World Medical Association Declaration of Helsinki, and the integrity procedures for scientific research involving human beings. Written informed consent was obtained from all participants prior to their participation in the study. The study was conducted over a three-month period. Inclusion criteria were the employment at USZ’s urology department, while exclusion criteria included neurological conditions (e.g., epilepsy). No financial compensation was provided, but participants had the opportunity to review their data and practice using the da Vinci Skills Simulator (dVSS).

### Materials

#### EEG- muse headband S - EEG - wireless EEG

**Fig. 1:**
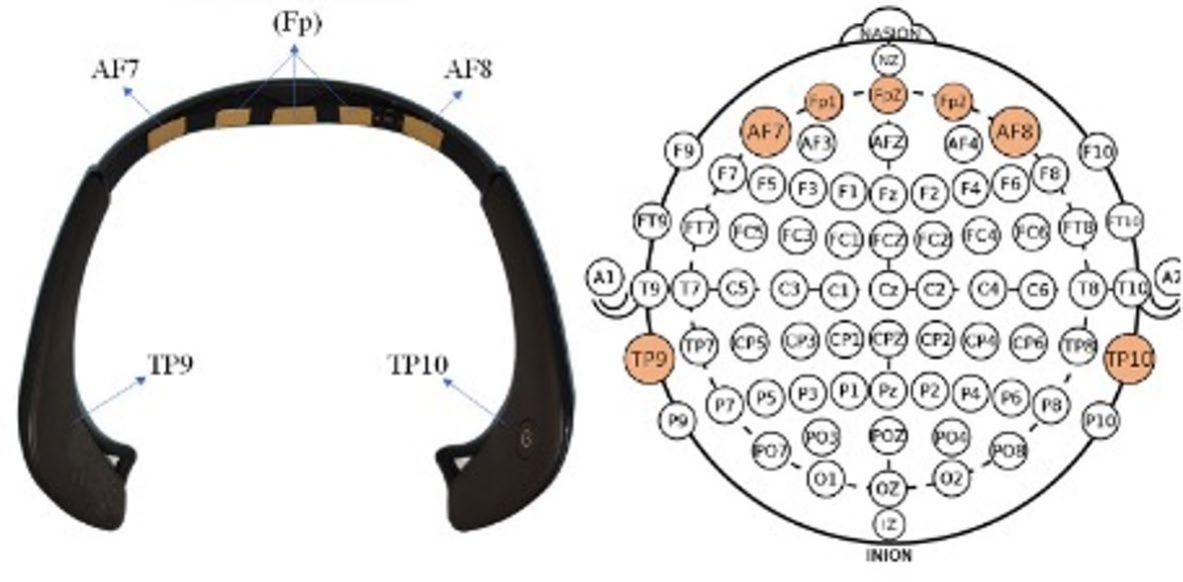
The Muse EEG headband. *Note.* The Muse S headband consists of five electrodes AF7, AF8, TP9, TP10 and a reference electrode (Fpz) (left). The electrodes are adapted from a conventional EEG, containing 10–20 EEG electrode placements by international standards. (right). Reprinted from Maula et al. ^32^.

The Muse S Headband (InteraXon Inc., Toronto, Canada) was used for wireless EEG monitoring in the operating theatre (Fig. 1). With a weight of 50 g, it requires no conductive gel and contacts the skin below the hairline. It includes five electrodes positioned at the frontal site on the forehead (namely, AF7 and AF8), at the temporal site (TP9 and TP10), as well as the reference electrode Fpz (front). The electrode placement follows the international 10–20 EEG standard. The sampling rate was 256 Hz with a 50 Hz notch filter to reduce power line interference.

#### Software

**Fig. 2:**
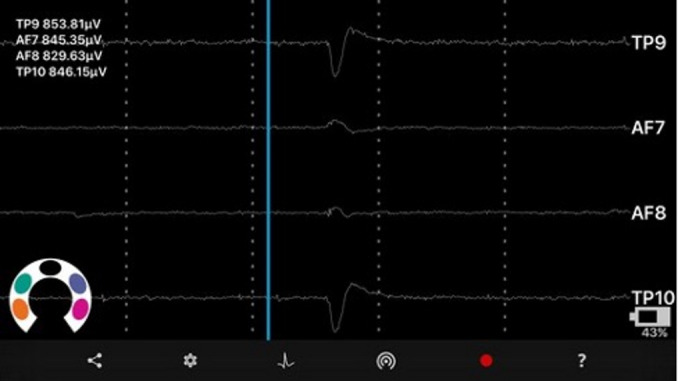
The mind monitor software recording the EEG. *Note*. A screenshot of the software Mind Monitor during an EEG recording. EEG is recorded among the electrodes TP9, AF7, AF8 and TP10 in µV.

The Mind Monitor software application (Fig. 2) was used for real-time EEG recording via Bluetooth with the Muse Headband. It captured raw EEG signals (0 to 1682 µV) across the four channels and processed them into five frequency bands: Delta (1–3 Hz), Theta (4–7 Hz), Alpha (8–13 Hz), Beta (13–30 Hz), and Gamma (> 30 Hz). The app utilised the Power Spectral Density (PSD) via Fast Fourier Transformation (FFT), displaying EEG values in a readable {0:100} range.

#### Da Vinci skills simulator for robotic-assisted surgery

**Fig. 3:**
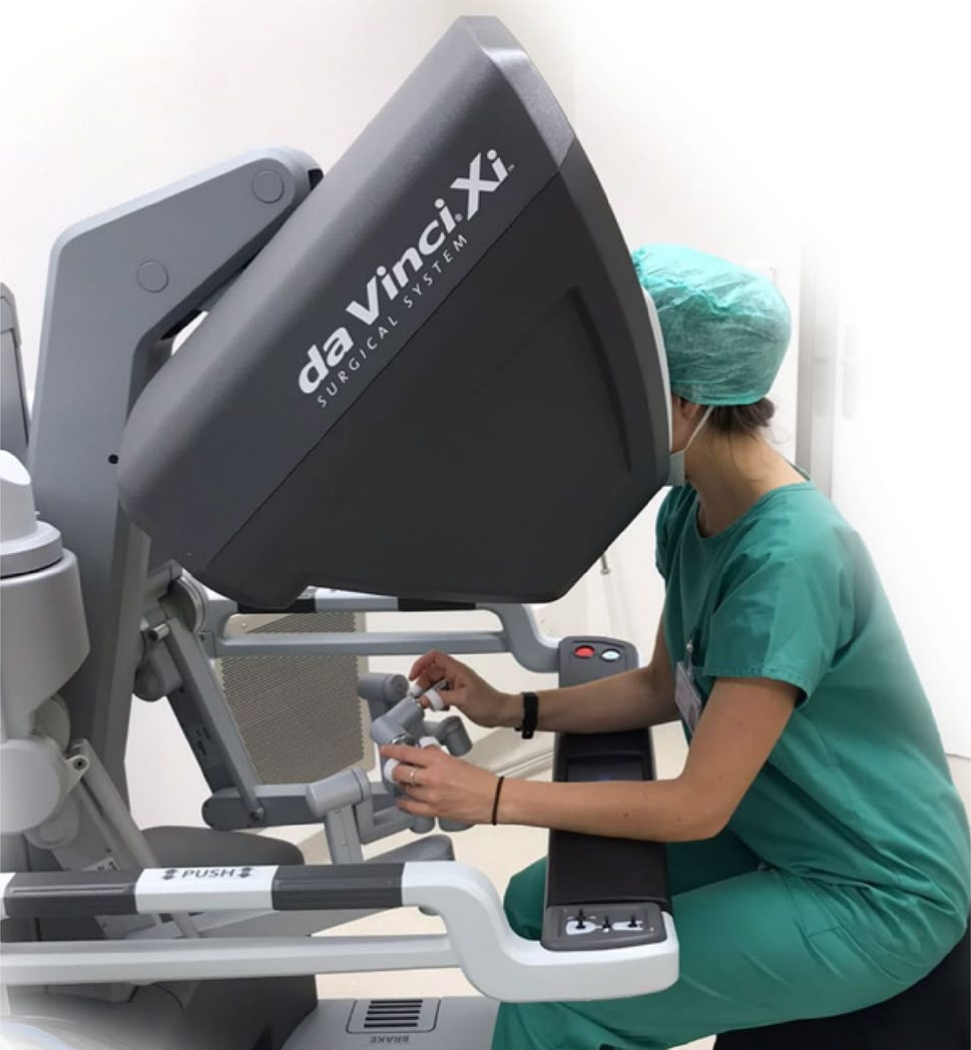
A da Vinci Skills Simulator during the experimental task. *Note*. A picture of a participant using the da Vinci Skills Simulator during the study.

The da Vinci Surgical System (Intuitive Surgical, Sunnyvale, CA, USA) is a robotic system for laparoscopic surgery, featuring four robotic arms, a 3D vision system, and EndoWrist instruments for enhanced range of motion (Fig. 3). The dVSS is an attachable computer that provides a VR environment for surgical training. It allows surgeons to practice motor coordination and procedures based on actual surgical operations in a 3D video environment.

#### Heart rate sensor

Heart rate (HR) in beats per minute (bpm) was measured using the Polar H10 chest strap, a wireless sensor connected via Bluetooth (Polar Electro Oy, Kempele, Finland). The Polar H10, designed to minimise electrical noise to ensure accurate ECG measurement, is reliable for HR analysis during various activities^26^. HR recording began with the EEG baseline and ended after the last Energy Dissection exercise. Data were collected using Polar Flow software.

#### Subjective flow data: the FSS questionnaire

The Flow Short Scale (FSS) was used post-task to measure subjective flow experience. It comprises 13 items rated on a 7-point Likert scale (1 = not true, 7 = true). The first dimension of the scale, including items one to ten (Cronbach’s α = 0.90), assesses smooth activity progression and absorption, while the remaining three items (Cronbach’s α = 0.80-0.90) measure general concerns and worries^1,27^ (see supplemental appendix).

### Experimental tasks

**Fig. 4:**
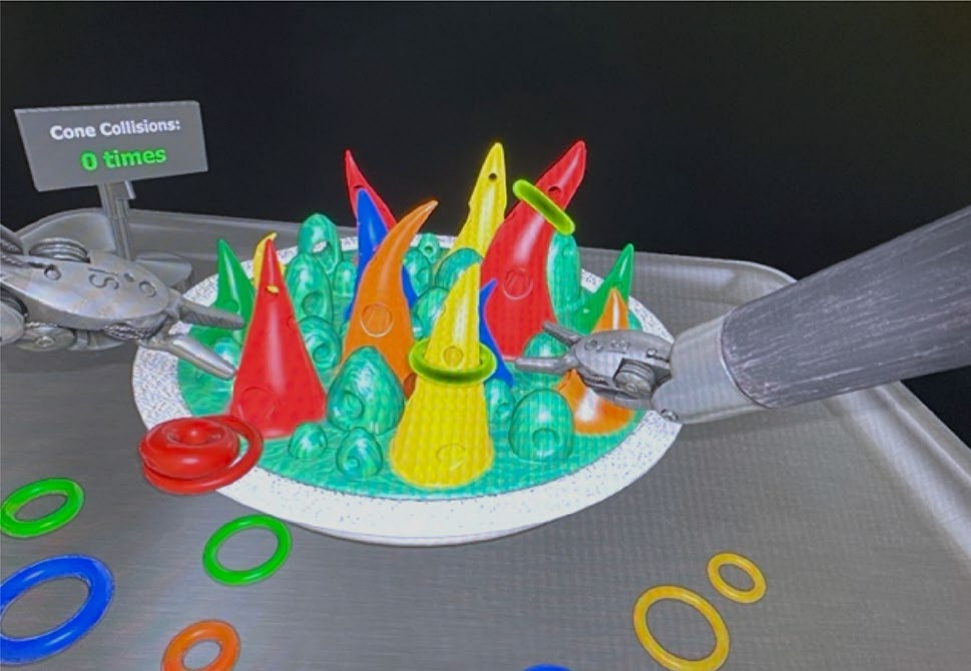
Experimental task 1: sea spikes. *Note.* Participants had to place the coloured rings on the matching coloured cones.

**Fig. 5:**
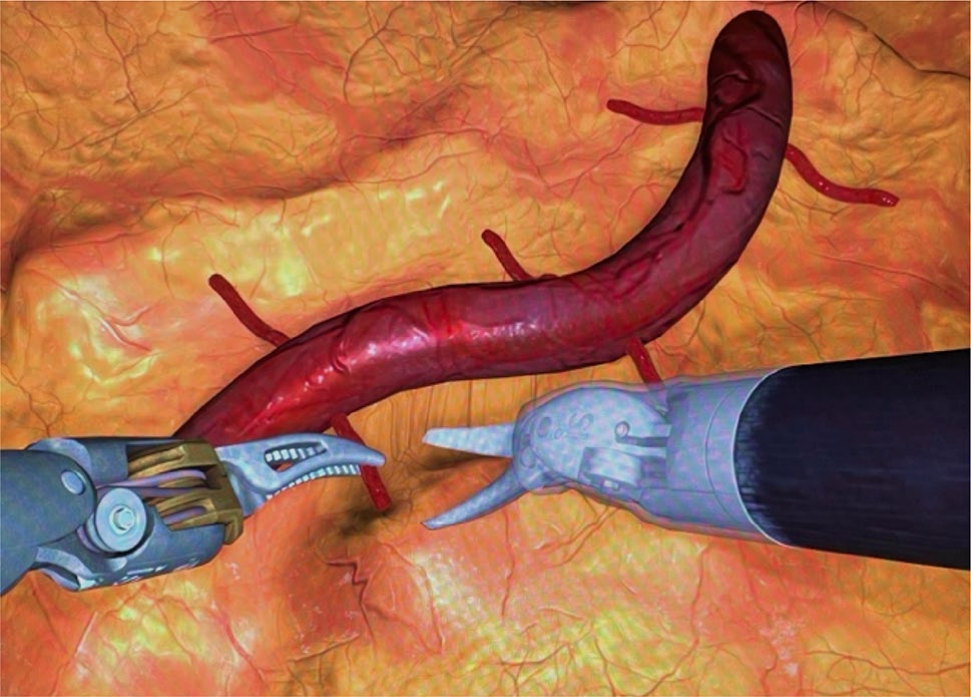
Experimental task 2: energy dissection. *Note.* Participants had to cut several vessels.

**Fig. 6:**
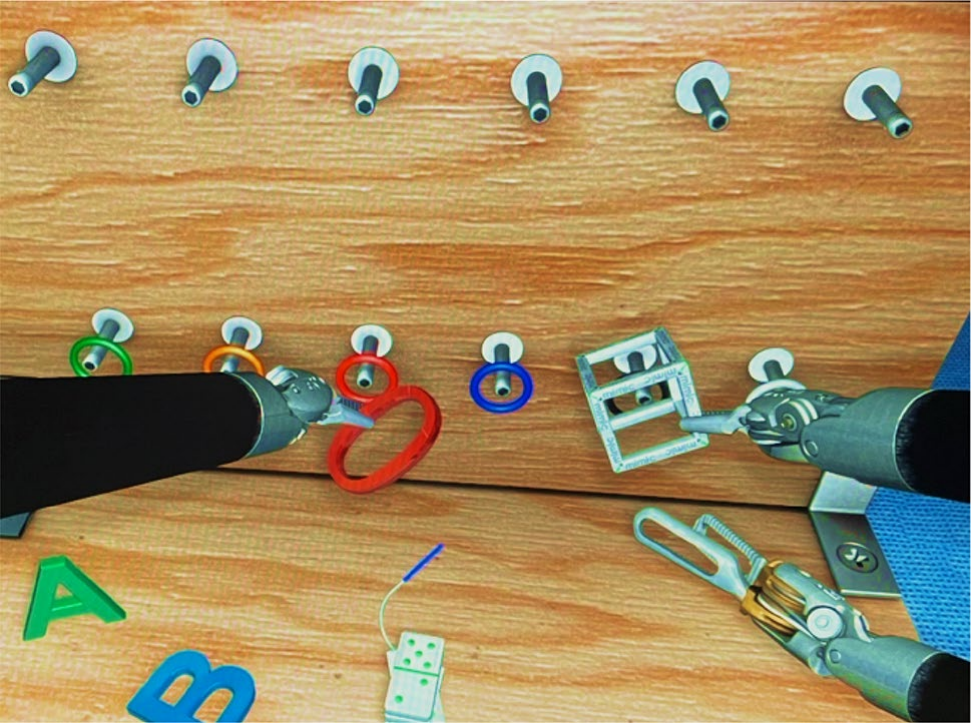
A participant’s view during the introductory exercise. *Note.* The introduction exercise gave the opportunity to test all basic functions.

The dVSS software includes training exercises for surgical procedures with the Da Vinci Console beforehand. Participants completed two VR exercises: “Sea Spikes,” (Fig. 4) a beginner task^28^, and “Energy Dissection,” (Fig. 5) an advanced task^29^. The score was calculated by considering the efficiency metrics of economy of motion and time to complete the exercise, along with penalties for instrument collisions, excessive instrument force, and instruments out of view. The introductory session allowed participants to familiarise themselves with the dVSS functions beforehand (Fig. 6).

#### Experimental task 1: sea spikes

The first task, “Sea Spikes,” is a beginner’s exercise where participants place coloured rings on matching cones as quickly and accurately as possible (Fig. 4).

#### Experimental task 2: energy dissection

“Energy Dissection” is a more advanced exercise involving additional functions like applying energy to cauterise bleeding vessels^29^. Performed after “Sea Spikes,” this task required participants to cauterise and cut smaller vessels to free a larger one. The footswitch panel allowed swapping between monopolar and bipolar energy instruments (Fig. 5).

### Experimental procedure

This study used a mixed design with between-subject and within-subject factors over three sessions, spaced 24 h to one week apart (details in supplemental Appendix).

In the USZ urologic operating room, participants changed into appropriate attire. The Polar H10 chest strap and Muse Headband were adjusted and tested. EEG baseline recordings were taken while participants sat still, alternating between eyes closed and open phases.

**Fig. 7:**
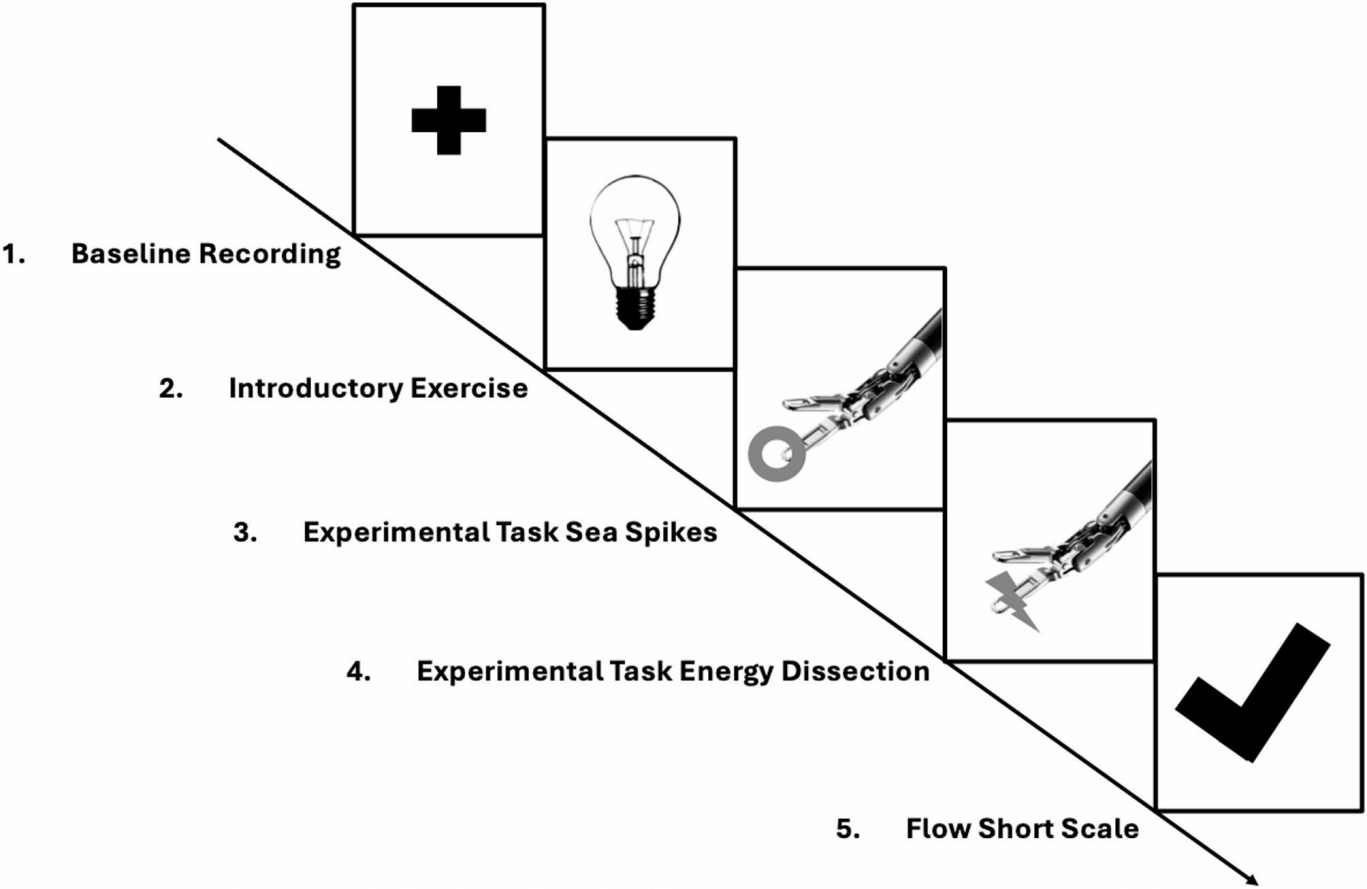
Study procedure of InFlow-opera. *Note.* Overview of the study procedure for the InFlow-Opera study, including baseline recordings, introductory exercise, experimental tasks, and the completion of the Flow Short Scale.

Participants used the dVSS, adjusting settings for comfort. They first completed an introductory exercise to familiarise themselves with the equipment. Next, they performed the “Sea Spikes” and “Energy Dissection” tasks, watching instructional videos beforehand. EEG and HR data were recorded throughout, starting with the first instrument movement and ending upon task completion. This procedure was repeated three times, with continuous HR recording (Fig. 7). After the second task, participants completed the FSS questionnaire.

### Data analysis

#### EEG data pre-processing

The raw EEG data was recorded at a sampling rate of ~ 256 Hz with a 10-bit resolution. The raw EEG signals were processed in MATLAB using the EEGLAB toolbox (Delorme & Makeig, 2004). Initially, the signals were band-pass filtered between 1 and 40 Hz to eliminate low-frequency drift and high-frequency noise.

EEG artefacts were addressed through Independent Component Analysis (ICA) using EEGLAB. ICA allows for the decomposition of EEG signals into independent components, making it easier to identify and remove artefacts related to non-neural activities, such as eye blinks, muscle tension, or external electrical noise. Components identified as artefacts, based on stereotypical patterns such as eye movements or muscle activity, were manually excluded from the data. Due to the suboptimal fit of nearly all female participants and the unstable connection to the temporal areas, the temporal electrodes TP9 and TP10 had to be entirely excluded from the analysis.

### Statistical analysis

Data consistency and normality of EEG PSD were assessed using Shapiro-Wilk tests. The Rank Sum (Mann-Whitney) test compared scores between groups. Generalized Estimating Equation (GEE) models with log gamma distribution analysed group differences over time for Theta Activity and HR. 95% confidence intervals were computed for means. All tests were two-sided, with p-values < 0.05 considered significant. Spearman’s rank correlation assessed relationships between Theta, FSS, experimental task Scores, and HR. Analyses were conducted using STATISTICA 13, PASW 24, and RStudio.

## Results

### Sample allocation to groups

Twenty surgeons were categorised based on their “Sea Spikes” scores, a beginner-level VR surgery task^28^. The average score was 174.45. Surgeons scoring above this threshold were classified as “High Performers” (13 surgeons; *M* = 30 years, 3 females, 10 males, *SD* = 4.3), and those scoring below as “Low Performers” (7 surgeons; *M* = 33 years, 2 females, 5 males, *SD* = 5.32). Age differences between Low Performers (*M* = 33 years, *SD* = 5.32) and High Performers (*M* = 30 years, *SD* = 4.3) were compared using an independent samples t-test. The results indicated no significant difference in mean ages between the two groups *t(*18) = -1.64, *p* = .119)

### Score comparison between groups

We examined the distribution of dependent variables (Theta, Scores, HR, and FSS) to determine performance disparities between High and Low Performers. Non-normality was confirmed using the Kolmogorov-Smirnov (KS) test (*D* = 0.112, *p* = .410), indicating a gamma distribution. Therefore, the two-sample Wilcoxon (Mann-Whitney U) test and Generalized Estimating Equation (GEE) models were applied. The Wald Chi-Squared Test assessed the significance of explanatory variables in the GEE models.

### Normality of score distributions

The Shapiro-Wilk test indicated that the scores were not normally distributed, *W* = 0.906, *p* < .001, rejecting the null hypothesis of normality at the conventional alpha level of 0.05.

### Mann-Whitney U test results

**Fig. 8:**
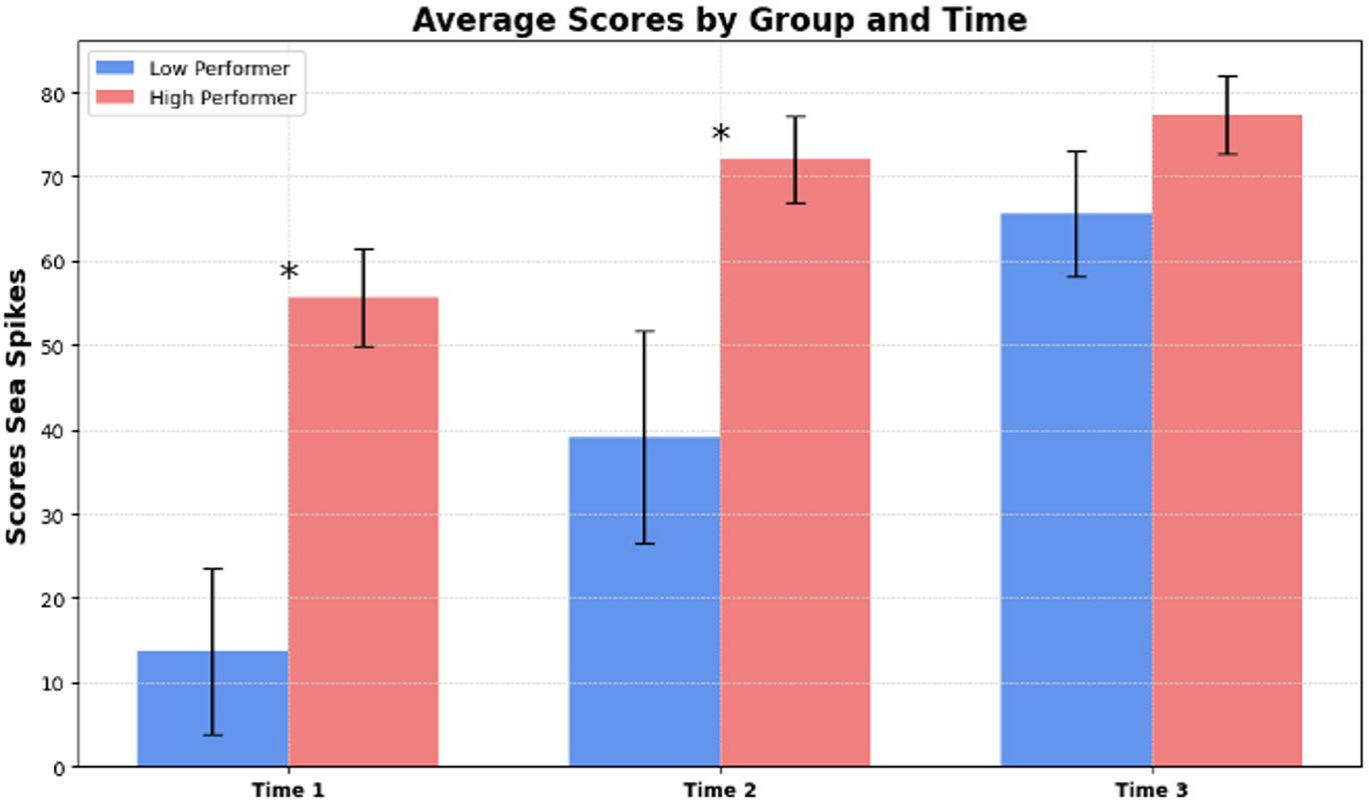
Group comparison of scores. *Note*. Comparison of scores between high and low performers across three time points during the “Sea Spikes” task. Significant differences were observed at Time points 1 and 2.

The Mann-Whitney U test was conducted to compare the scores of two groups at three different time points (Fig. 8). The results were as follows :

- Time point 1: A significant difference in scores was found between the groups, *U* = 14.0, *p* = .013.
- Time point 2: The scores again differed significantly, *U* = 17.0, *p* = .026.
- Time point 3: The difference in scores was not statistically significant, *U* = 31.0, *p* = .267.

### Generalised eEstimation equation models

The Wald Chi-Squared Test was used to determine the significance of explanatory variables in the model. The model tested the contribution of the variables time and group effects (Low Performer vs. High Performer) in the Sea Spikes exercise.

### Theta activity in sea spikes exercise among electrode AF8

Comparing the results of the Model for the dependent Variable Theta Activity in electrode AF8, a significant difference in Theta activity over the three times measurements was found. The Model was significant in group differences with *Wald*(1) = 5.961, *p* = .015 with a significant effect over time with *Wald*(2) = 16.534, *p* < .001 but no interaction effect with *Wald*(2) = 3.254, *p* = .197.

**Fig. 9:**
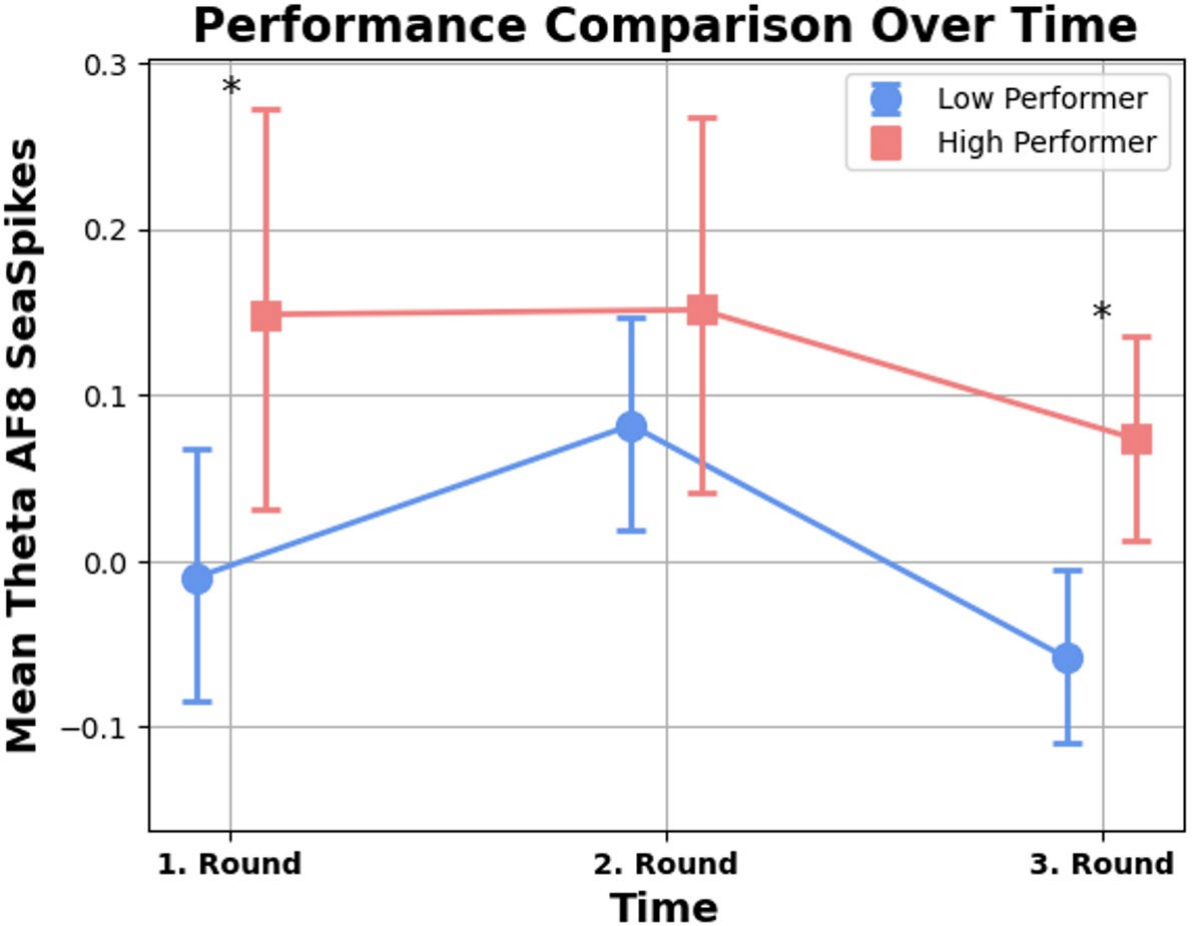
Group comparison of sea spikes performance and mean Theta AF8. *Note*. Group comparison showing performance scores and mean Theta activity at electrode AF8 during the “Sea Spikes” task. High performers exhibited significantly higher Theta activity.

A post hoc pairwise comparison was performed to identify the variables that determined the significance of the Theta differences among the three time measurements and the groups in the model (Table 1). The pairwise comparison revealed that the average Theta of High performers was about 0.159 higher (*MD* = 12.10, *SE* = 0.073) than the average Theta of Low Performers during the first round with *p* = .029 (Fig. 9). Similar findings were observed during the third measurement: High Performers had on average 0.132 higher Theta activity than Low Performers (*MD* = 0.132, *SE* = 0.041) during the third experimental session with *p* = .001(Fig. 9).

**Table 1:** Pairwise comparison of sea spikes task.

Scores over time pairwise comparison		Mean Difference	Std. Error	Sig.	95% Wald confidence interval for difference	
					Lower	Upper
Time 1 * Low Performer	Time 1 * High Performer	-0.159	0.073	0.029*	-0.301	-0.016
Time 2 * Low Performer	Time 2 * High Performer	-0.069	0.067	0.296	-0.200	0.061
Time 3 * Low Performer	Time 3 * High Performer	-0.132	0.041	0.001*	-0.213	-0.051

Significant values are in [bold].

Note. Pairwise comparison of Sea Spikes task performance and mean Theta activity across time points.

### Theta activity in energy dissection exercise among electrode AF8

Comparing the results of the Model for the dependent Variable Theta Activity in electrode AF8 during the Energy Dissection Exercise, a significant difference in Theta activity over the three experimental measurements was found. The Model was significant in group differences with *Wald*(1) = 5.11, *p* = .002 with a significant effect over time with *Wald*(2) = 10.59, *p* = .005 but no interaction effect with *Wald*(2) = 6.838, *p* = .311.

**Fig. 10:**
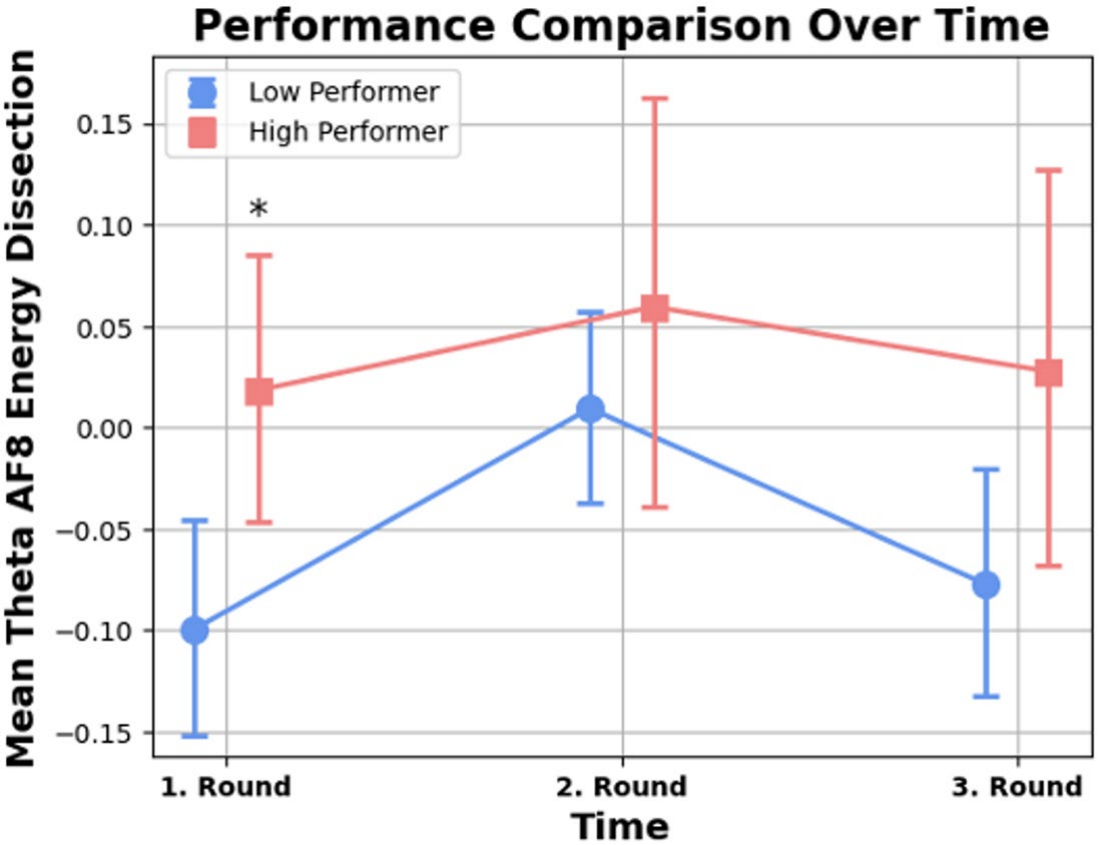
Group comparison of energy dissection performance and mean Theta AF8. *Note*. Comparison of performance scores and mean Theta activity at electrode AF8 during the “Energy Dissection” task. Significant group differences were observed in the first round.

A post hoc pairwise comparison was performed to identify what variables were determining the significance of the Theta differences among the three-time measurements and the groups in the model (Table 2). The pairwise comparison revealed that the average Theta of High performers was about 0.118 higher (*MD* = 0.118, *SE* = 0.043) than the average Theta of Low Performers during the first round with *p* = .006. (Fig. 10). No significant differences were found in rounds 2 and 3.

**Table 2:** Pairwise comparison energy dissection task.

Scores over time pairwise comparison		Mean difference	Std. error	Sig.	95% Wald confidence interval for difference	
					Lower	Upper
Time 1 * Low Performer	Time 1 * High Performer	-0.118	0.043	0.006*	-0.202	-0.033
Time 2 * Low Performer	Time 2 * High Performer	-0.050	0.057	0.379	-0.162	0.062
Time 3 * Low Performer	Time 3 * High Performer	-0.105	0.057	0.068	-0.217	0.008

Significant values are in [bold].

Note. Pairwise comparison of Energy Dissection task performance and mean Theta activity across time points.

### Heart rate data

No significant difference in minimal heart rate was found between low and high performers with *Wald*(1) = 0.465, *p* = .495. Also, no significant group differences were found in average heart rat with *Wald*(1) = 0.347, *p* = .056 and maximum heart rate with *Wald*(1) = 1.259, *p* = .267.

### Correlations

**Fig. 11:**
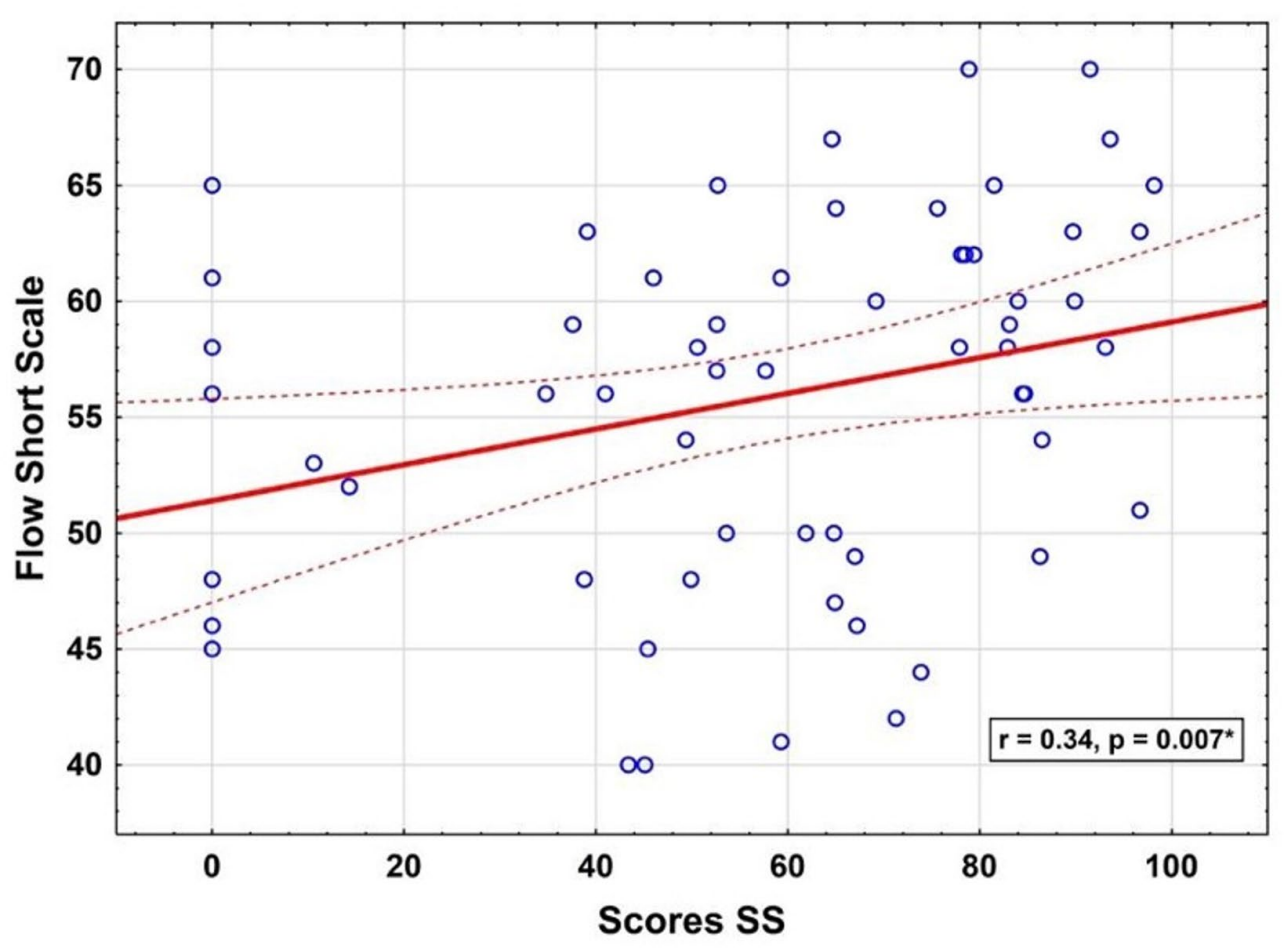
Flow short scale and sea spikes score. *Note.* The figure visualises the significant positive correlation between the scores achieved in Sea Spikes (Scores SS) and the Flow Short Scale.

The variables FSS scores, Theta activity, age of participants, performance scores, and HR were analysed for potential correlations. A positive correlation emerged between the performance scores achieved during ‘Sea Spikes’ exercises and the subjective Flow experience in Dimension One among all participants (Fig. 11), *r*(58) = 0.341, *p* = .007, considered a strong effect^30^.

## Discussion

The present study aimed to explore the neurological and physiological correlates of the flow state in urologic surgeons during virtual robotic-assisted surgical tasks, specifically examining Theta activity and HR recordings.

One significant observation was the increased Theta activity in the right electrode (AF8) in the high-performer group compared to the low-performer group during two surgical tasks. To the best of our knowledge, this is the first time, that the neurological association of flow state with increased performance could be measured in surgeons performing surgical tasks on the dVSS. Due to the fact that the dVSS is identical to the console used for actual operations, the scenario closely resembles actual surgical practice^31^. By adopting this setting and allowing the participants to immerse in the described tasks, it was possible to show that a higher Theta activity indeed correlates with better performance scores, as reported in studies investigating high-performance environments^13,14^.

The heightened Theta activity at AF8 corresponding to the right side of the forehead (anterior frontal region)^32^ indicates enhanced cognitive control and attentional engagement. This lateralisation can be attributed to the right hemisphere’s role, particularly the right prefrontal cortex, in maintaining attention, cognitive control and visuospatial processing^33^, which are crucial for complex surgical tasks^34^. These findings align with the study of Katahire et al.^8^, which found increased Theta activities in frontal brain areas related to a high level of cognitive control and the immersion aspect of flow. Several other studies that investigated the neurophysiological basis of the flow experience confirm these findings by demonstrating heightened activation in the lateral prefrontal cortex and reduced activation in the amygdala during flow state under difficult task settings, as opposed to feelings of boredom or overwhelm^35–37^.

Considering the difference in Theta activity in the “Sea Spikes” task observed in the first and third rounds between the two groups, but not in the second, suggests a potential combined learning curve and fatigue effect resulting in a U-shaped representation of the Theta activity differences between the high performer and low performer group over time as proposed in the paper by Adadayoobi et al.^38^. During the first round, participants might be highly engaged as they familiarise themselves with the task^39^. The third round might reflect a consolidation of skills and deeper engagement^39^, whereas the second round could represent a transition phase where the initial novelty wears off, leading to a temporary decrease in engagement or fatigue effect^38^. This pattern in our data is supported by previous research indicating that Theta waves are associated with high engagement and cognitive control^9,10^. The absence of significant differences in the second round might reflect a stabilisation period before full engagement is re-established in the third round. Additionally, the scores did not differ significantly between the second and the third rounds, suggesting a plateau in performance improvement typical for surgical learning curves, potentially due to task familiarity or reduced novelty^39^.

In the second experimental task; energy dissection, the lack of significant differences in the second and third rounds could also be due to familiarity with the dVSS and the setup, as it represented the last task in the study sequence after the participants were already exposed to the training exercise and the SS task, for the second and the third time reducing cognitive load temporarily, resulting in a decreased flow state^40^. An alternative explanation could be a decreasing motivation and/or decreasing competitive spirit as the surgeons might have already displayed in Task 1 their self-perceived best performance. Similar explanation has been discussed for example in a study of mindset of elite athletes^41^.

Interestingly, no significant differences in heart rate were found between the high and low performers. This could be attributed to the absence of true baseline HR measurements due to the experimental setup, which is a notable limitation. Baseline HR is crucial for accurate comparison, as it provides a reference point for assessing changes due to task engagement. Without it, interpreting HR differences is challenging. Additionally, HR may not be as sensitive as EEG measures in detecting subtle variations in flow-related cognitive and emotional states^18,19^.

The positive correlation between the Flow Short Scale (FSS) scores and performance in the “Sea Spikes” task across all groups highlights the subjective experience of flow and its impact on performance in various domains^42,43^. This correlation suggests that higher perceived flow is associated with better task performance, reinforcing the concept that flow enhances cognitive and motor functions necessary for complex tasks^44^.

Several limitations must be acknowledged. First, the exclusion of the temporal electrodes TP9 and TP10 due to poor fit and unstable connection, particularly in female participants. This exclusion may have affected the completeness of the EEG data recording; controversially, we were able to detect significant differences in Theta activity between the groups with our limited experimental setup; this could, therefore, also be an indication that relevant EEG research could also be carried out with just a mono-channel EEG, as proposed by Anwar et al.^45^. Second, the absence of baseline HR measurements limits the interpretation of HR data. Future studies should include baseline measurements to comprehensively understand physiological changes associated with flow. Additionally, the small sample size and the focus on just two types of surgical tasks may limit the generalizability of the findings. Expanding the study to include EEG recordings during real operations, along with a larger sample size, would yield more robust data. Additionally, incorporating more channels could help identify broader patterns and track how Theta activity increases over time using Event-Related Spectral Perturbation analysis, making these steps the logical progression for future research.

## Conclusions

This study provides specific data for the association of the flow state and increased performance in robotic surgeons, adding to the growing field of flow state and performance research. Acquiring a better understanding of the neural and physiological mechanisms of flow in this context could provide the basis for neurofeedback-enhanced training protocols and Theta activity as a new biomarker to evaluate training progress. Ultimately, robotic surgeon candidates may not be evaluated solely on their performance scores. Instead, incorporating their corresponding Theta activity could provide a more reliable predictor for learning curve progression. This approach may lead to better patient outcomes and reduced stress levels among surgical professionals.

## Supplementary Information

Below is the link to the electronic supplementary material.

Supplementary Material 1

## Data Availability

All codes and raw data supporting the findings of this study are available at : 10.17605/OSF.IO/ZKG2V. These data include all relevant materials collected during the research and can be accessed via this link.
