# Supplementary material for: Investigation of flow state occurrence during robotic virtual reality operations
Source: Sci Rep. 2025 Nov 19;15:40710. doi: 10.1038/s41598-025-24215-0 (PMC12630671; doi:10.1038/s41598-025-24215-0)
Supplement: Supplementary file 1 — Supplementary Material 1 [file 41598_2025_24215_MOESM1_ESM.docx]

**Investigation Of Flow State Occurrence During Virtual**

**Reality Operations**

**Flow Short Scale**

**Experimental Procedure**

The dVSS was placed in the urologic operation room at the hospital, and participants were instructed to change clothes according to the operating theatre rules. At the beginning of every session, the Polar H10 chest strap was individually adjusted, and the recording of the HR was tested. Additionally, the Muse Headband was individually adjusted, and the electrode connection was checked with the Mind Monitor application’s impedance check. In addition, the dVSS was individually adjusted according to personal ergonomic preferences.

Shortly after adjusting all devices, the baseline recording was conducted before starting the experimental tasks. During the baseline recording, participants were asked to sit in front of a fixation cross on one wall of the operation theatre. They were instructed to keep their head, jaw and body still and relaxed. Simultaneously with the EEG baseline recording, the HR was recorded from these time stamps contentiously until the experimental task was over. In the first sixty seconds of the EEG baseline recording, participants were asked to close their eyes and relax their bodies and faces. In the second part of the baseline recording, also lasting sixty seconds, participants were asked to open their eyes and look at the fixation cross 1.5 meters in front of them. The baseline recordings were directly sent as a CSV file to the Hospital Cloud-Network via the Mind Monitor software. Baseline recordings were used for correction phases in the analysis and to check if a typical decrease in alpha activity was measurable between the eyes closed and eyes open condition. After recording the baseline, participants changed to the dVSS. Ergonomics settings were adjusted according to height and view through the visual system as well as the size of the finger cuffs for the instrument handling.

After personalizing the ergonomic settings, the introductory exercise *playground* was started on the simulator for 120 seconds. Participants were instructed to use all the instruments, using the view pedal to adjust their field of vision. After 120 seconds of reaccustoming, the first experimental exercise, *Sea Spikes* started. Participants were instructed to watch the explanatory video with the instructions for the exercise. After the instruction video and the start of the exercise, no talking or asking questions to the experimenter were allowed. The EEG recording started when the participant made the first movement with an instrument in the VR environment and stopped when the task was marked as completed. No maximum duration was predefined for the task. After completing the first exercise Sea Spikes, the experiment was directly continued with the second exercise, Energy Dissection. Participants were instructed to watch the specific instruction video. After the instruction video and the start of the exercise, no talking or asking questions to the experimenter was allowed. The EEG recording started when the participant made the first movement with an instrument in the VR environment and stopped when the task was marked as completed. No maximum duration was predefined for the task. Additionally, the HR recording was stopped. After completing the tasks, participants removed the HR sensor as well as the EEG and filled out the FSS questionnaire.

This procedure was repeated three times, in the exact same way, on three different days, with a maximum time frame of one week between each session. In difference to the EEG data, HR was recorded permanently during the baseline, the Sea Spikes exercise, and the Energy Dissection exercise without separating between conditions as done with the EEG data. Due to the technical set up of the Polar H10 software and belt, stopping the recording for each condition and starting it new would have needed a manual new positioning of the HR belt as well as new login to the software. Due to the tight schedule of the surgeons and limited, timely resources, the whole session was recorded without interruption.
